# Use of Seprafilm in a Patient With Recurrent Small Bowel Obstruction: A Case Report

**DOI:** 10.7759/cureus.28910

**Published:** 2022-09-07

**Authors:** Yitong Xiao, Dale A Calixte, Erin M Kenney, Elizabeth Fry, Frederick Tiesenga

**Affiliations:** 1 Surgery, Saint James School of Medicine, Chicago, USA; 2 Surgery, St George’s University School of Medicine, Chicago, USA; 3 General Surgery, West Suburban Medical Center, Chicago, USA

**Keywords:** surgical retreatment, adhesions, umbilical hernia repair, seprafilm, small bowel obstruction

## Abstract

Small bowel obstruction (SBO) is an emergency that should be managed early to reduce the risk of bowel perforation, strangulation and subsequent life-threatening organ dysfunction caused by sepsis. A prompt diagnostic workup including imaging and lab studies is required to assess the severity of the obstruction and to establish if emergency surgery is required. We report the case of a 55-year-old male with a history of recurrent SBO, previous ventral hernia repair and indications of previous colonic tubular adenoma per colonoscopy findings. The patient underwent an exploratory laparotomy procedure and adhesiolysis to release the SBO. This case report emphasizes the safety and efficacy of Seprafilm placement intraoperatively in decreasing the occurrence of postoperative adhesions in abdominal laparotomy procedures.

## Introduction

Small bowel obstruction (SBO) is a partial or complete blockage of the small intestine causing buildup of waste and gas proximal to the obstruction. SBO is a common medical emergency, with 350,000 admissions per year [1]. Common presenting symptoms of a complete obstruction include abdominal pain/distention, nausea, vomiting, and constipation [2]. In the adult population, the leading cause of SBO is postoperative adhesions. Other common etiologies include incarcerated hernias, malignancy, and inflammatory bowel disease [2]. 

Imaging studies, such as abdominal x-ray or CT scans, are important in the diagnosis of SBO. An abdominal x-ray can show dilated loops of bowel while a CT scan may show a transition point. Once SBO is identified, treatment includes IV fluids and bowel rest with NPO. Bowel decompression via a nasogastric (NG) tube may also be used to alleviate symptoms. If the intestine is completely obstructed or strangulated, surgery may be required [3]. Prevention of postoperative SBO via adhesion barrier is indicated for patients undergoing abdominal or pelvic laparotomy. Seprafilm is made up of sodium hyaluronate (HA) and carboxymethylcellulose (CMC) placed intraoperatively to form a barrier between the abdominal wall and the underlying viscera [3]. Importantly, a meta-analysis showed that the use of seprafilm decreases the incidence and severity of postoperative adhesions [4]. This case study reviews the use of Seprafilm for an SBO patient with a history of recurrent SBO, and previous ventral hernia repair and stresses the importance of preventing SBO when possible [5].

## Case presentation

**Figure 1:**
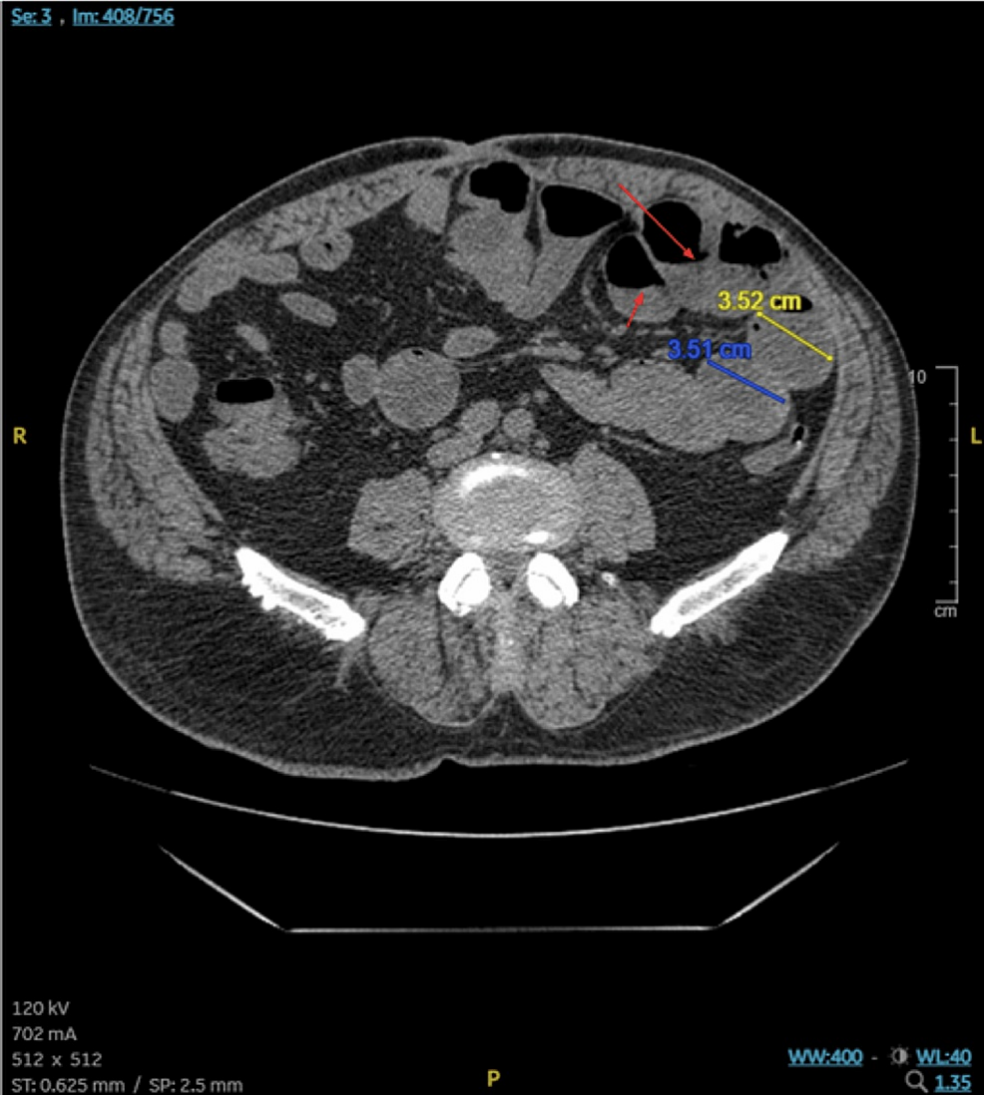
CT scan of the abdomen Remarkable for small bowel obstruction with dilated loops of small bowel measuring up to 3.5cm (examples indicated by yellow and blue lines with respective measurements). Fluid levels are seen within the colon (red arrows).

A 55-year-old Caucasian male presented to the emergency room with abdominal pain, nausea, vomiting and diarrhea for three days. His abdominal pain was constant without any precipitating or alleviating factors. The patient also reported positional vertigo for the past two days. He denied any blood in his emesis or bowel movement, fevers, and sick contacts. The patient has been admitted to the hospital for observation due to SBOs multiple times in the past. Beyond recurrent SBO, the patient’s past medical history is remarkable for coronary heart disease, type 2 diabetes mellitus, hypertension, and hyperlipidemia. Past surgical history includes umbilical hernia repair, esophagogastroduodenoscopy and colonoscopy. The patient’s home medication regimen includes Aspirin, Atorvastatin, Lantus, Norco, insulin lispro, lidocaine topical, lisinopril and metformin. Based on the patient's medical history and presenting symptoms, differentials included gastroenteritis, SBO, and diabetic ketoacidosis (DKA). Labs obtained at triage demonstrate leukocytosis to 11.1, glucose in the 600s without evidence of DKA. Physical exam findings included abdominal distension with hyperactive bowel sounds. CT of the abdomen and pelvis without contrast was remarkable for SBO (Figure 1).

**Figure 2:**
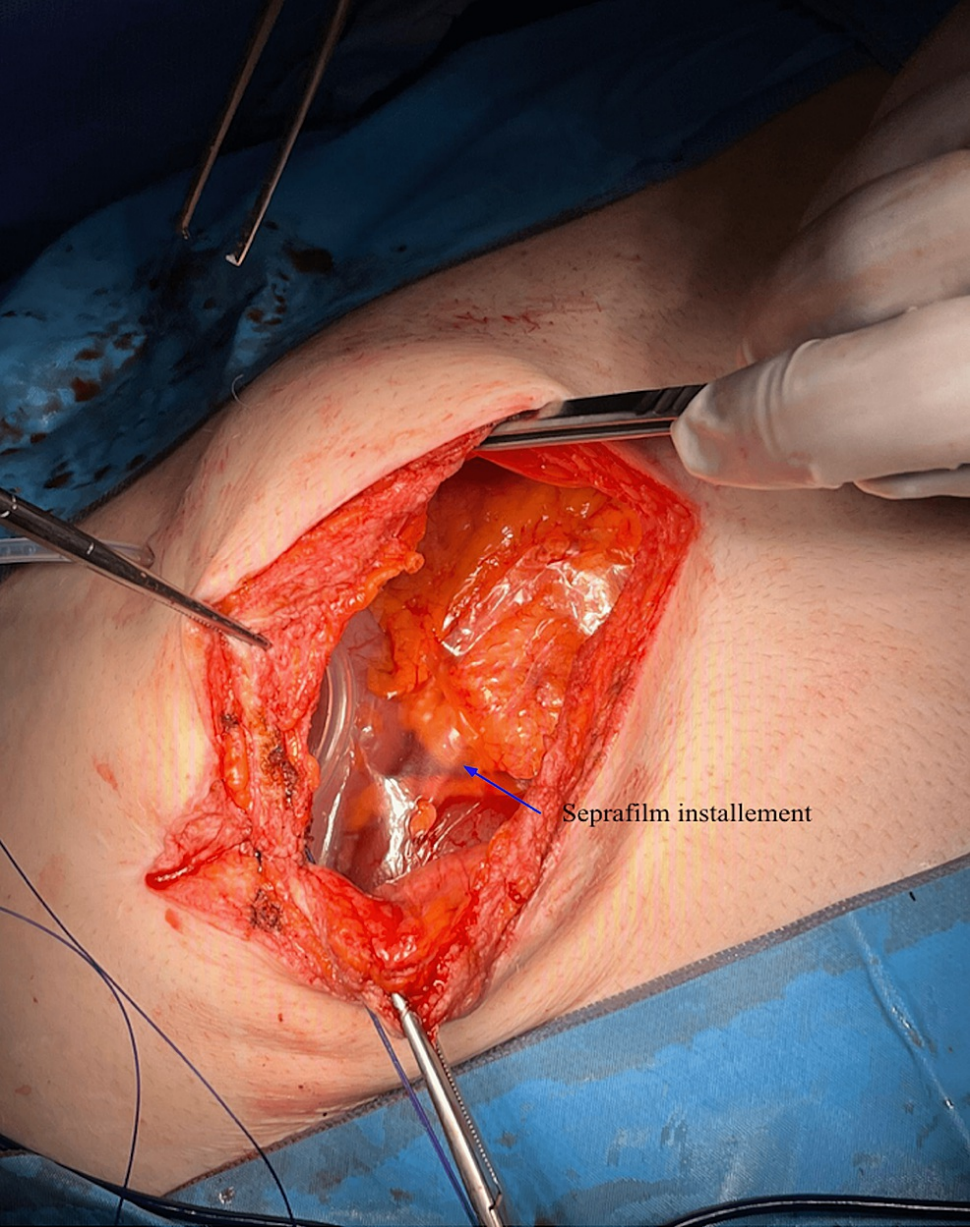
Exploratory laparotomy. Seprafilm installation (blue arrow).

The patient was admitted to the hospital and due to high suspicion of recurrent SBO, an NPO order and NG tube insertion were recommended to the patient. The patient refused the initial recommendation for NG tube placement. The patient was informed about the suspected SBO and risks and benefits of surgery. He was also informed that proceeding with surgery would require placement of an NG tube under anesthesia, which the patient consented to. Exploratory laparotomy was performed under general anesthesia. The patient was opened through a midline incision. Upon entering the peritoneal cavity, there were multiple loops of massively dilated small bowel with collapsed small bowel distally. The small bowel was also stuck to the anterior abdominal wall at the site of the previous midline incision. This was carefully excised using Metzenbaum scissors. Adhesiolysis was applied to free the small bowel loops from themselves, completely releasing the obstruction. At this time, meticulous hemostasis was assured. Antibiotic irrigation was utilized and retrieved. Jackson-Pratt surgical drain was left in the abdomen and brought out through a separate stab incision. Considering the patient's past surgical history and recurrence of bowel obstruction, we decided to utilize the Seprafilm to create a barrier between intestines, the previous mesh from the patient's ventral hernia repair and abdominal wall to prevent future tissue adhesion (Figure 2).

The patient was maintained on a small bowel rest regimen, deep vein thrombosis (DVT) prophylaxis, and antibiotics for 24 hours postoperatively. Bowel functions started convalescing on postoperative day 2. Upon normal serial abdominal x-ray and return of bowel functions, the patient was discharged.

## Discussion

SBO remains a fundamental cause of morbidity and financial cost in many hospitals worldwide [6]. Among the major causes, adhesions remain the culminant etiology of SBO at a rate of 74% [7]. Adhesions may be acquired or congenital; however, most acquired adhesions are a result of previous abdomino-pelvic surgery. Less commonly, adhesions may result from inflammatory conditions, intraperitoneal infection or abdominal trauma. Although the extent of adhesion formations varies greatly from one patient to another and by the magnitude of the surgery performed, other surgical factors have demonstrated to contribute to adhesion formation such as mesh, glove powder, suture material and spilled gallstones [8]. These surgical factors can trigger a plethora of problems and cause significant morbidity and mortality.

The cause of our patient’s SBO was due to his history of abdominal surgeries, including umbilical hernia repair, esophagogastroduodenoscopy, and colonoscopy. The decision to use Seprafilm was made based on our patient’s past medical history of recurrent SBO with adhesions. The goal of using Seprefilm in this case was to prevent further adhesions from the exploratory laparotomy, as well as, to offer insight to other clinicians in managing patients with similar SBO presentation. Seprafilm is a bioresorbable membrane composed of chemically modified sodium HA and CMC, which is placed intraoperatively to form a barrier between the abdominal wall and the underlying viscera to help reduce surgical post adhesions between the abdominal wall and other abdominal structures such as the small bowel [9].

Multiple clinical research studies have been conducted to evaluate the beneficial outcome of Seprafilm application in preventing adhesive lesions and recurrent bowel obstruction in the past 15 years. In a study conducted by Fazio et al. [10], 1,701 patients were given Seprafilm after undergoing an intestinal resection procedure. The incidence of adhesive SBO requiring reoperation was significantly lower for Seprafilm patients compared with the control. Salum et al. studied 191 patients with loop ileostomy construction after applying Seprafilm compared to a control group. The results indicated that Seprafilm significantly decreased adhesion formation around the stoma without any increase in the need for myotomy or enterotomy [11]. Based on a systematic review and meta-analysis from Zeng et al., Seprafilm could potentially decrease abdominal adhesions after abdominal surgery, which may benefit patients [12]. However, this study pointed out that Seprafilm could not reduce postoperative intestinal obstruction. At the same time, Seprafilm can increase the risk for abdominal abscesses and anastomotic leaks. Further longitudinal studies are needed to assess the effectiveness and value of Seprafilm in preventing bowel obstruction and enhancing healthcare team outcomes.

## Conclusions

SBO has become a common medical issue that can usually be treated with conservative therapies such as bowel rest and NG tube decompression. Surgery may be needed if these therapies fail to resolve the obstruction. The leading cause of SBO is adhesions from previous abdominal surgeries. This patient, a 55-year-old Caucasian male, came to the emergency department with signs and symptoms of SBO. The patient had a past surgical history of umbilical hernia repair, esophagogastroduodenoscopy and colonoscopy. This patient also reported recurrent SBO but refused initial NG tube placement and was considered a good candidate for an exploratory laparotomy to resolve the obstruction. In the operating room, there were extensive adhesions that required meticulous and labor-intensive care to extricate. Seprafilm was placed during surgery as precaution against further SBO. Seprafilm can be used to minimize the frequency and severity of SBO from abdominal surgeries, to hopefully avoid the complications that were seen in this patient from his previous umbilical hernia repair.
